# Deletion of *Runx2* in Articular Chondrocytes Decelerates the Progression of DMM-Induced Osteoarthritis in Adult Mice

**DOI:** 10.1038/s41598-017-02490-w

**Published:** 2017-05-24

**Authors:** Lifan Liao, Shanxing Zhang, Jianhong Gu, Takeshi Takarada, Yukio Yoneda, Jian Huang, Lan Zhao, Chun-do Oh, Jun Li, Baoli Wang, Meiqing Wang, Di Chen

**Affiliations:** 1 Department of Orthopedic Surgery, Rush University Medical Center, Chicago, IL 60612 USA; 2 State Key Laboratory of Military Stomatology, Department of Oral Anatomy and Physiology and TMD, School of Stomatology, Fourth Military Medical University, Xi’an, 710032 China; 3 Institute of Orthopaedics and Traumatology, Zhejiang Chinese Medical University, Hangzhou, 310053 China; 4 College of Veterinary Medicine, Yangzhou University, Yangzhou, 225009 China; 5 Department of Regenerative Science, Okayama University Graduate School of Medicine, Dentistry and Pharmaceutical Sciences, 2-5-1 Shikata-cho, Kita-ku, Okayama 700-8558 Japan; 6 Section of Prophylactic Pharmacology, Venture Business Laboratory, Kanazawa University Kakuma-machi, Kanazawa, Ishikawa, 920-1192 Japan; 7 Department of Medical Cell Biology and Genetics, Shenzhen Key Laboratory and the Center for Anti-Ageing and Regenerative Medicine, Shenzhen University Medical School, Shenzhen, 518060 China; 8 Key Lab of Hormones and Development (Ministry of Health), Tianjin Key Lab of Metabolic Diseases, Tianjin Medical University, Tianjin, 300070 China

## Abstract

Runx2 may play an important role in development of osteoarthritis (OA). However, the specific role of Runx2 in articular chondrocyte function and in OA development in adult mice has not been fully defined. In this study, we performed the destabilization of the medial meniscus (DMM) surgery at 12-week-old mice to induce OA in adult *Runx2* ^*Agc1CreER*^ mice, in which *Runx2* was specifically deleted in *Aggrecan*-expressing chondrocytes by administering tamoxifen at 8-weeks of age. Knee joint samples were collected 8- and 12-weeks post-surgery and analyzed through histology, histomorphometry and micro-computed tomography (μCT). Our results showed that severe OA-like defects were observed after DMM surgery in Cre-negative control mice, including articular cartilage degradation and subchondral sclerosis, while the defects were significantly ameliorated in *Runx2* ^*Agc1CreER*^ KO mice. Immunohistochemical (IHC) results showed significantly reduced expression of MMP13 in *Runx2* ^*Agc1CreER*^ KO mice compared to that in Cre-negative control mice. Results of quantitative reverse-transcription PCR (qRT-PCR) demonstrated that expression of the genes encoding for matrix degradation enzymes was significantly decreased in *Runx2* ^*Agc1CreER*^ KO mice. Thus, our findings suggest that inhibition of Runx2 in chondrocytes could at least partially rescue DMM-induced OA-like defects in adult mice.

## Introduction

Osteoarthritis (OA) is the most common degenerative joint disease, affecting close to 27 million Americans. The major pathological features of OA include progressive loss of articular cartilage, osteophyte formation, the increases in subchondral bone mass and synovial tissue inflammation and hyperplasia^1, 2^. Multiple animal models have been established to mimic the development of OA. Among them, DMM-induced OA is the most widely used OA animal model^3^.

During OA progression, articular chondrocytes undergo hypertrophic differentiation. Runx2 plays a pivotal role in regulation of genes important for chondrocyte differentiation, matrix degradation and osteoblast differentiation^4, 5^. Several studies reported that Runx2 expression levels are high in human OA cartilage^6–8^. To explore the role of Runx2 in OA progression, genetic animal models have been used. Mice with *Runx2* overexpression (*Runx2-Tg)* display an increased number of cartilage protease expression in chondrocytes^9^. Overexpression of *Runx2* also activates matrix degradation enzymes (MMP13 and ADAMTS5) through mitogen-activated protein kinase (MAPK) pathways^10^ and through direct regulation of *Mmp13* gene transcription^11^. Previous work in our laboratory showed that deletion of *Tgfbr2* in chondrocytes up-regulates Runx2 and accelerates OA progression^12^. A recent study reported that Runx2 has been identified as a novel potential target of miR-105 and the FGF2-p65-miR-105-Runx2 axis might play an important role in OA pathogenesis^13^. A previous study reported that heterozygous *Runx2* global KO mice exhibited decreased cartilage destruction and osteophyte formation after induction of knee joint instability^14^. Since mice used in this study is global *Runx2* heterozygous KO mice (*Runx2* ^+/−^), the specific effect of Runx2 on articular cartilage in adult mice remains undefined.

Aggrecan is a major extracellular matrix protein in articular cartilage. *Aggrecan* gene is expressed more robustly than *Col2a1* gene, another cartilage matrix component in adult mice^15, 16^. To determine the role of *Runx2* in OA development in adult mice, we have generated *Runx2* ^*Agc1CreER*^ conditional KO mice by crossing *Runx2* ^*flox/flox*^ mice^17^ with *Agc1-CreER* transgenic mice^16^. In the present studies, we determined if *Runx2* specific deletion in chondrocytes in adult mice has chondro-protective effect on DMM-induced OA development.

Matrix metalloproteinase 13 (MMP13) is a potent enzyme that targets cartilage for degradation. MMP13 expression was low in normal and early degenerative cartilage but was strongly up-regulated in late-stage OA specimens^18^. Moreover, transgenic mice with constitutively active MMP13 expression in the hyaline cartilages and joints developed pathological changes in articular cartilage of mouse joints similar to those observed in human OA^19^. Clinical investigation suggests that MMP13 may be associated with cartilage degradation during OA development^20^. This clinical observation was further confirmed by the study with *Mmp13* KO mice. Pharmacological inhibition of MMP13 activity has been demonstrated to be an effective strategy to decelerate articular cartilage loss in a murine model of injury-induced knee OA^21^.

The relationship of Runx2 and *Mmp13* has been studied in the developing skeleton during the process of endochondral ossification. The DNA sequence of Runx2 binding site was originally described as osteoblast-specific elemenet-2 (OSE2), which is essential for expression of osteoblast-specific gene osteocalscin^22, 23^. The *Mmp13* proximal promoter contains an OSE2 site conserved among different species, such as human, rabbit, mouse and rats^24–27, 11^. Runx2 binds to the OSE2 site in the *Mmp13* promoter and increases *Mmp13* gene transcription in cooperation with c-Fos and c-Jun binding to a neighboring AP-1 site^26, 28–30^. Moreover, co-transfection of Runx2 with the *Mmp13* promoter in osteosarcoma UMR 106–01 cells has been shown to enhance *Mmp13* promoter activity^29^. It has been showed that 148 bp upstream of *Mmp13* transcription start site is sufficient and necessary for *Mmp13* gene expression in bone, teeth and skin *in vivo* and the AP-1 and Runx2 binding sites are likely to regulate this *Mmp13* proximal promoter activity. *Runx2* also regulates *Mmp13* during chondrocytes differentiation. A recent study reported that the interaction of Runx2 and Osterix, a downstream molecule of Runx2, cooperatively induces *Mmp13* expression during chondrocyte differentiation^31^. In recent studies, we demonstrated, through mutation analysis and ChIP assays, that Runx2 activates *Mmp13* expression by binding to the OSE2 site located in the proximal region of the human *Mmp13* promoter in chondrocytes^11^.

## Results

### *Agc1-CreER* mice drive Cre recombination in articular chondrocytes

**Figure 1:**
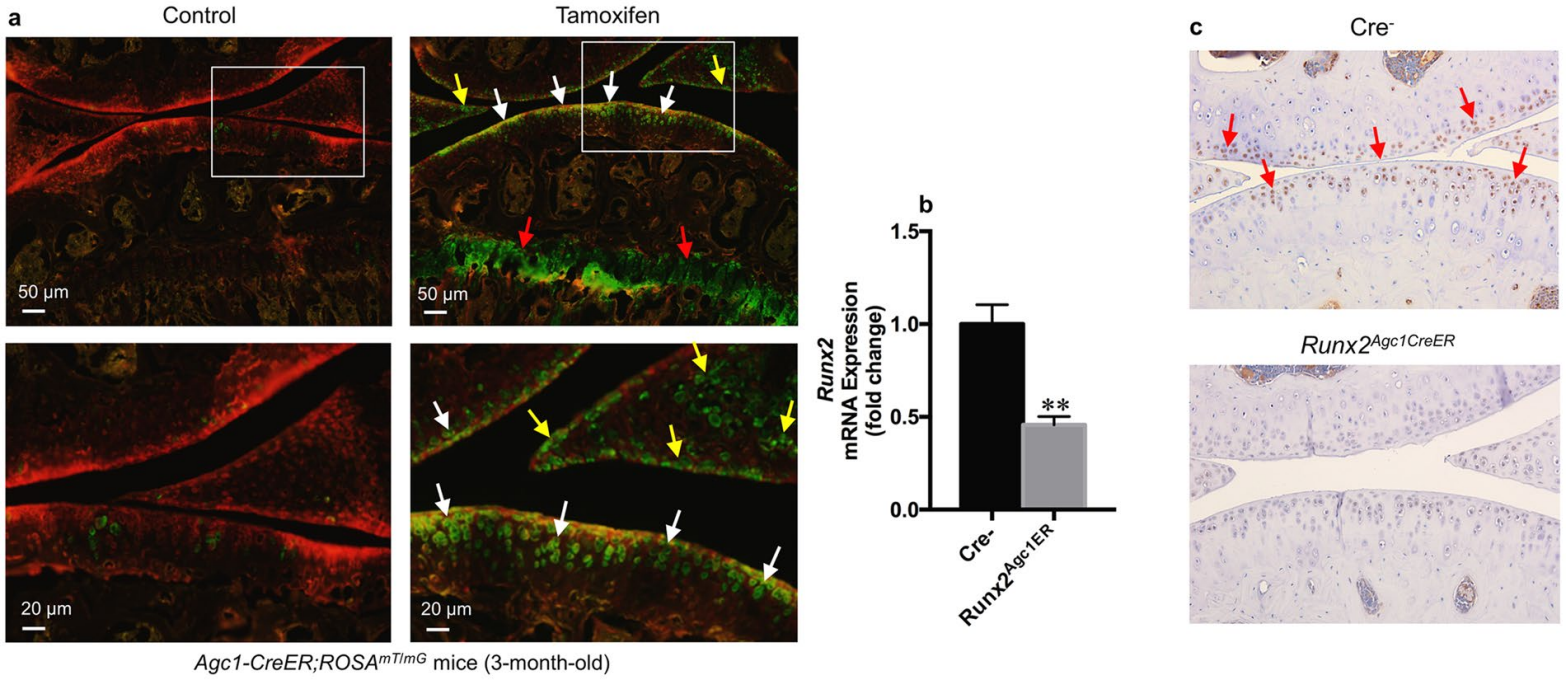
Directed Cre recombination in articular chondrocytes from *Agc1-CreER* mice. (**a**) *Agc1-CreER* mice target articular chondrocytes in adult mice. *Agc1-CreER; ROSA* ^*mT/mG*^ mice were generated by breeding *Agc1-CreER* transgenic mice with *ROSA* ^*mT/mG*^ reporter mice. Tamoxifen or vehicle control was administered into 2-month-old *Agc1-CreER; ROSA* ^*mT/mG*^ mice. Bone samples were harvested from 3-month-old mice after they were injected with tamoxifen at age of 2 months (1 mg/10 g body weight, i.p. injection, daily for 5 days). Histologic sections of *Agc1-CreER; ROSA* ^*mT/mG*^ mice with or without tamoxifen treatment were analyzed by fluorescence microscopy. The results showed that *Agc1-CreER* targeting GFP-positive cells (green color cells) are located in growth plate (red arrowheads), articular cartilage (white arrowheads) and meniscus (yellow arrowheads) in *Agc1-CreER; ROSA* ^*mT/mG*^ mice. (**b**) Significant decrease in *Runx2* mRNA expression was observed in *Runx2* ^*Agc1CreER*^ mice compared to their Cre-negative littermates. Total RNA was isolated from articular cartilage of 5-month-old *Runx2* ^*Agc1CreER*^ mice and their Cre-negative littermates and real-time PCR assay was performed. All mice were administrated with tamoxifen at 2-months of age (**P < 0.01 versus Cre-negative mice, unpaired Student’s *t*-test; n = 3 mice per group). (**c**) Immunohistochemical (IHC) results showed that Runx2 protein levels were significantly decreased in articular cartilage of *Runx2* conditional KO mice compared to Cre-negative mice (Red arrowheads show Runx2 positive cells, n = 3 mice per group).

To efficiently target articular chondrocytes in adult mice, we used *Agc1-CreER* transgenic mice^16^. We evaluated the targeting efficiency and specificity of these mice by breeding them with *ROSA* ^*mT*/*mG*^ reporter mice. *ROSA* ^*mT/mG*^ mice are double-fluorescent Cre reporter mice that express membrane-targeted tandem dimer Tomato (mT) prior to Cre-mediated excision and membrane-targeted green fluorescent protein (mG) after excision. Also, mG labeling is Cre-dependent, complementary to mT at single cell resolution, and distinguishable by fluorescence-activated cell sorting: red before and green after recombination^32^. Analysis of histologic frozen sections from 3-month-old mice (tamoxifen was given to the mice at 2-month-old) using fluorescence microscopy showed that *Agc1-CreER* targeting cells are located in growth plate, articular cartilage and meniscus in *Agc1-CreER; ROSA* ^*mT/mG*^ mice (Fig. 1a).

### The efficiency of *Runx2* KO in the articular cartilage in *Runx2*^*Agc1CreER*^ mice

To assess the efficiency of *Runx2* KO in the articular cartilage in adult *Runx2* ^*Agc1CreER*^ KO mice, we administrated tamoxifen to 2-month-old *Runx2* ^*Agc1CreER*^ mice and their Cre-negative littermates. We then sacrificed them at the 5-months of age. Subsequently, the q RT-PCR and IHC staining were performed. The q RT-PCR analysis showed that *Runx2* mRNA levels were significantly decreased by 60% in articular cartilage of *Runx2* ^*Agc1CreER*^ KO mice compared to Cre-negative control mice (Fig. 1b). Consistent with this result, the IHC analysis showed that Runx2 protein levels were decreased in articular cartilage of *Runx2* ^*Agc1CreER*^ KO mice (Fig. 1c).

### OA progression was decelerated in *Runx2*^*Agc1CreER*^ mice

**Figure 2:**
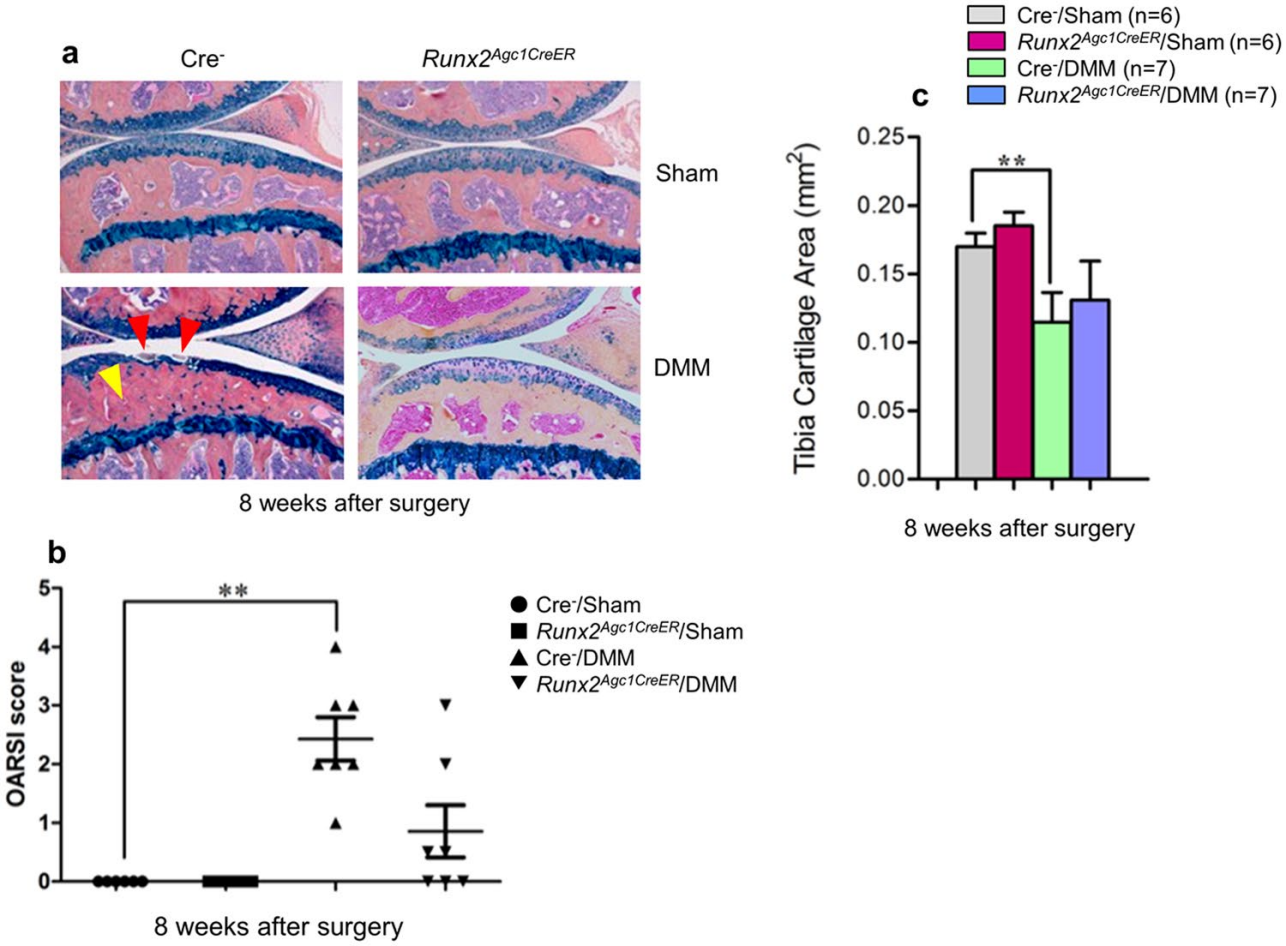
OA progression was decelerated in *Runx2* ^*Agc1CreER*^ mice 8 weeks after DMM surgery. Tamoxifen was administered into 8-week-old male Cre-negative control and *Runx2* ^*Agc1CreER*^ KO mice (1 mg/10 g body weight, i. p. injection, daily for 5 days). DMM surgery or Sham operation was performed when these mice were 12-week-old (right hind limbs). (**a**) Knee joints were harvested from Cre-negative and *Runx2* ^*Agc1CreER*^ KO mice 8 weeks post-surgery and Alcian blue/Hematoxylin Orange G staining was performed. Histological results showed that articular cartilage degradation (red arrowheads) and subchondral sclerosis (yellow arrowhead) were observed in Cre-negative control mice after DMM surgery. In contrast, defects in articular cartilage degradation and subchondral sclerosis induced by DMM surgery was significantly protected in *Runx2* ^*Agc1CreER*^ KO mice. (**b**,**c**) Histological sections were analyzed by OARSI scoring system and by histomorphometric method. The severity of OA-like phenotype was analyzed by grading histological sections using OARSI score system by two blinded observers. Articular cartilage area of tibia plateau was quantified by tracing the Alcian blue-positive staining areas using the OsteoMeasure system. These results demonstrated that DMM surgery caused significant OA-like defects in Cre-negative control mice. However, no significant difference, rather a decreased tendency of cartilage degeneration was observed in the mice 8-weeks after DMM surgery in *Runx2* ^*Agc1CreER*^ KO mice compared to Cre-negative control mice. (**P < 0.01, one-way ANOVA followed by Tukey’s post-hoc test; n = 6–7 mice per group).

**Figure 3:**
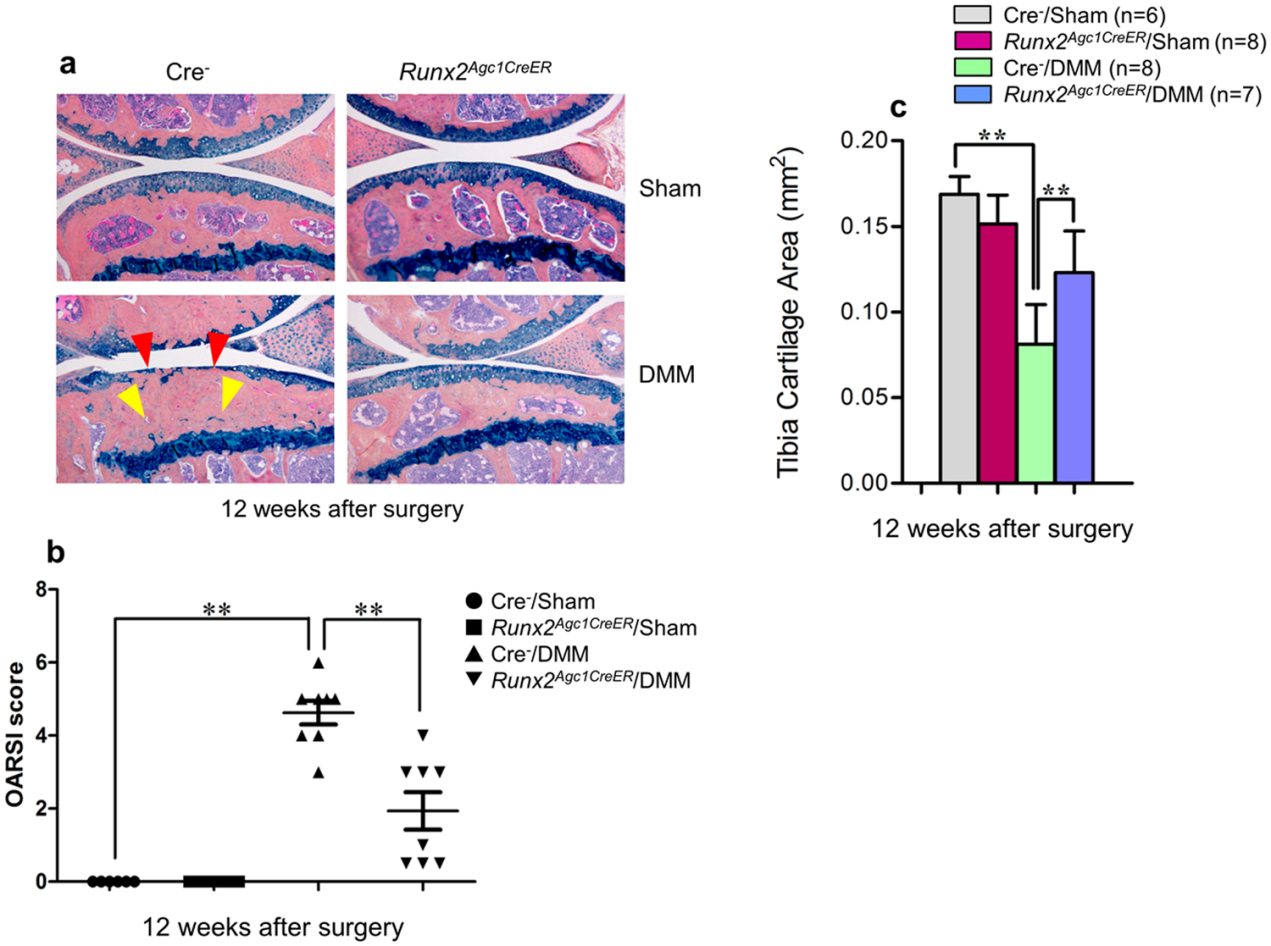
OA progression was protected in *Runx2* ^*Agc1CreER*^ mice 12 weeks after DMM surgery. (**a**) Knee joints were harvested from Cre-negative and *Runx2* ^*Agc1CreER*^ KO mice 12 weeks post-surgery and Alcian blue/Hematoxylin Orange G staining was performed. Histological results showed that articular cartilage degradation (red arrowheads) and subchondral sclerosis (yellow arrowheads) were observed in Cre-negative control mice after DMM surgery. In contrast, defects in articular cartilage degradation and subchondral sclerosis induced by DMM surgery was significantly protected in *Runx2* ^*Agc1CreER*^ mice. (**b**) Histological sections were analyzed by OARSI scoring system. The severity of OA-like phenotype was graded by OARSI scoring system by two blinded observers. The results demonstrated that DMM surgery caused significant OA-like defects in Cre-negative control mice. In contrast, DMM-induced OA-like defects (12-weeks post-surgery) were significantly protected in *Runx2* ^*Agc1CreER*^ KO mice (**P < 0.01, one-way ANOVA followed by Tukey’s post-hoc test; n = 6–8 mice per group). (**c**) Histological sections were analyzed by histomorphometric method. Articular cartilage area of tibia plateau was quantified by tracing the Alcian blue-positive staining areas using the OsteoMeasure system. DMM surgery led to significant loss of articular cartilage in Cre-negative control mice. In contrast, this effect was significantly protected by deletion of *Runx2* observed in *Runx2* ^*Agc1CreER*^ KO mice (12-weeks after DMM surgery) (**P < 0.01, one-way ANOVA followed by Tukey’s post-hoc test; n = 6–8 mice per group).

To investigate if *Runx2* deletion could prevent or decelerate DMM-induced OA-like defects, we crossed *Runx2* ^*flox/flox*^ mice with *Agc1-CreER* transgenic mice^16^ to generate the *Runx2* ^*Agc1CreER*^ conditional KO mice. Tamoxifen was administered into 8-week-old *Runx2* ^*Agc1CreER*^ mice. Deletion of *Runx2* had no significant effect on chondrocyte morphology (Figs 2a and 3a). To create an OA mouse model, DMM surgery was performed when the mice were 12-week-old. Knee joint samples were harvested 8- and 12-weeks post-surgery (n = 6–8 mice per group). Results of histological analysis showed that the OA-like phenotype, including fibrillation, clefting and cartilage degradation, was observed 8-weeks after DMM surgery and worsened at the 12-week time point in Cre-negative control mice. In contrast, much less articular cartilage excavation (but not statistical significant) was observed in 8-week-old *Runx2* ^*AgcCre1ER*^ KO mice (Fig. 2a). In contrast, deletion of *Runx2* significantly protected DMM-induced OA development at the time point 12-weeks after DMM surgery (Fig. 3a).

**Figure 4:**
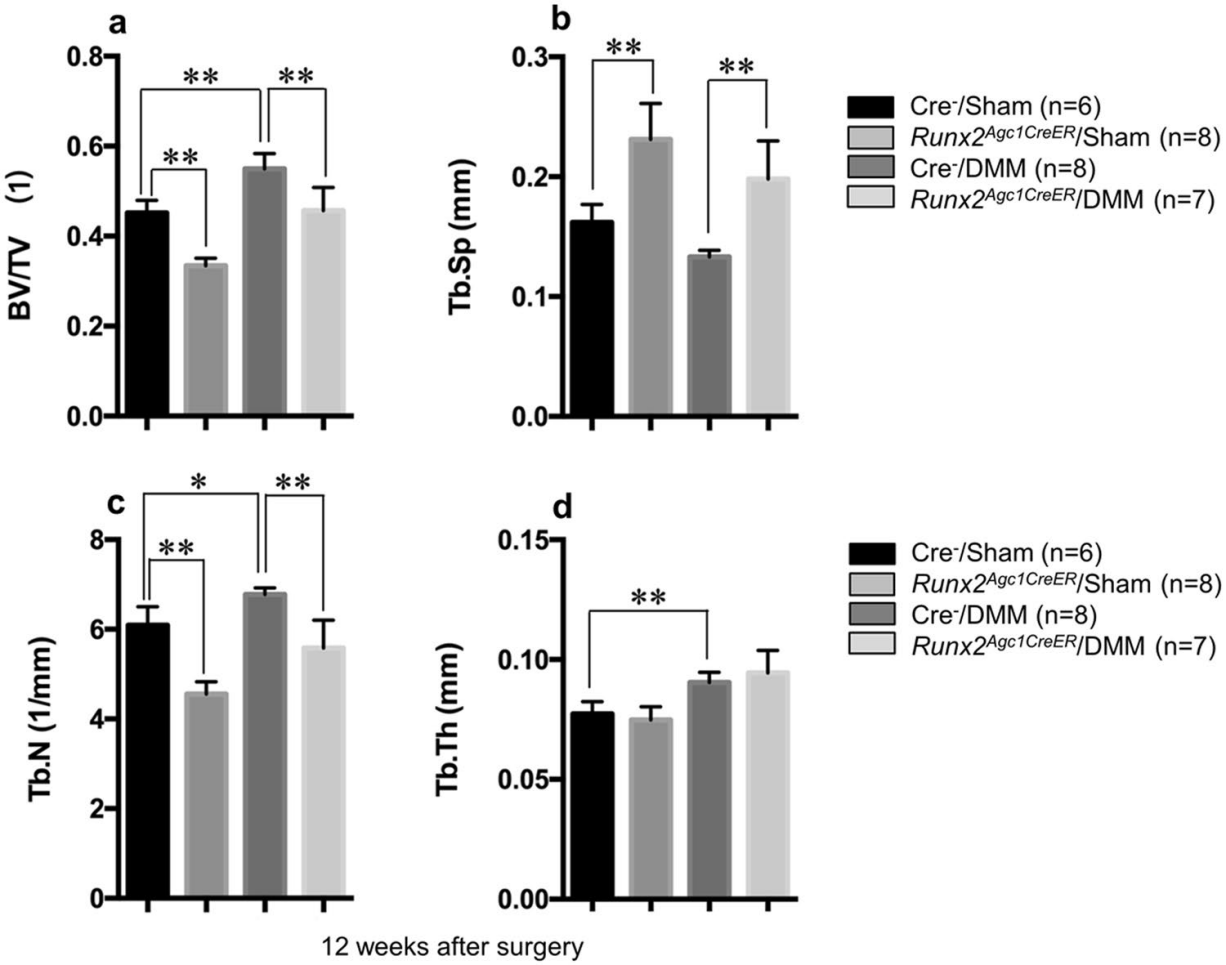
Micro-CT data display evidence of significantly increased bone mass of subchondral bone of knee joint in Cre negative mice with DMM surgery compared with Cre negative mice with sham surgery, and the increased bone mass was significantly reduced in *Runx2* ^*Agc1CreER*^ KO mice. All the mice were administrated tamoxifen at 2-months of age, followed by the DMM surgery performed at 3-months of age. Mice were sacrificed 3 months after surgery. (*P < 0.05, **P < 0.01, one-way ANOVA followed by Tukey’s post-hoc test; n = 6–8 mice per group).

The evaluation using OARSI scoring system revealed that there was no significant difference, rather a decreased tendency of cartilage degeneration in the mice 8-weeks after DMM surgery in *Runx2* ^*Agc1CreER*^ KO mice (Fig. 2b). With time progression, significantly reduced cartilage degeneration was observed in *Runx2* ^*Agc1CreER*^ KO mice 12-weeks after DMM surgery (Fig. 3b). We then quantify the OA progression by performing histomorphometry using the OsteoMeasure system. The results showed that at the time point 8-weeks after DMM surgery, although there was no significant difference between the *Runx2* ^*Agc1CreER*^ KO mice and Cre-negative controls, the tendency of increased articular cartilage area was observed in *Runx2* ^*Agc1CreER*^ KO mice (Fig. 2c). At the time point of 12-weeks after DMM surgery, deletion of *Runx2* significantly protected DMM-induced cartilage degradation (Fig. 3c). The subchondral sclerosis was observed 8- and 12-weeks after DMM surgery in Cre-negative control mice and this phenotype was also rescued in the *Runx2* ^*Agc1CreER*^ KO mice (Figs 2a and 3a). Consistent with this result, the data of μCT analysis showed that subchondral bone mass was increased in Cre-negative control mice 12-weeks after DMM surgery and this effect was rescued in the *Runx2* ^*Agc1CreER*^ KO mice (Fig. 4).

### The increased MMP13 protein levels by DMM surgery were rescued by *Runx2* deletion

**Figure 5:**
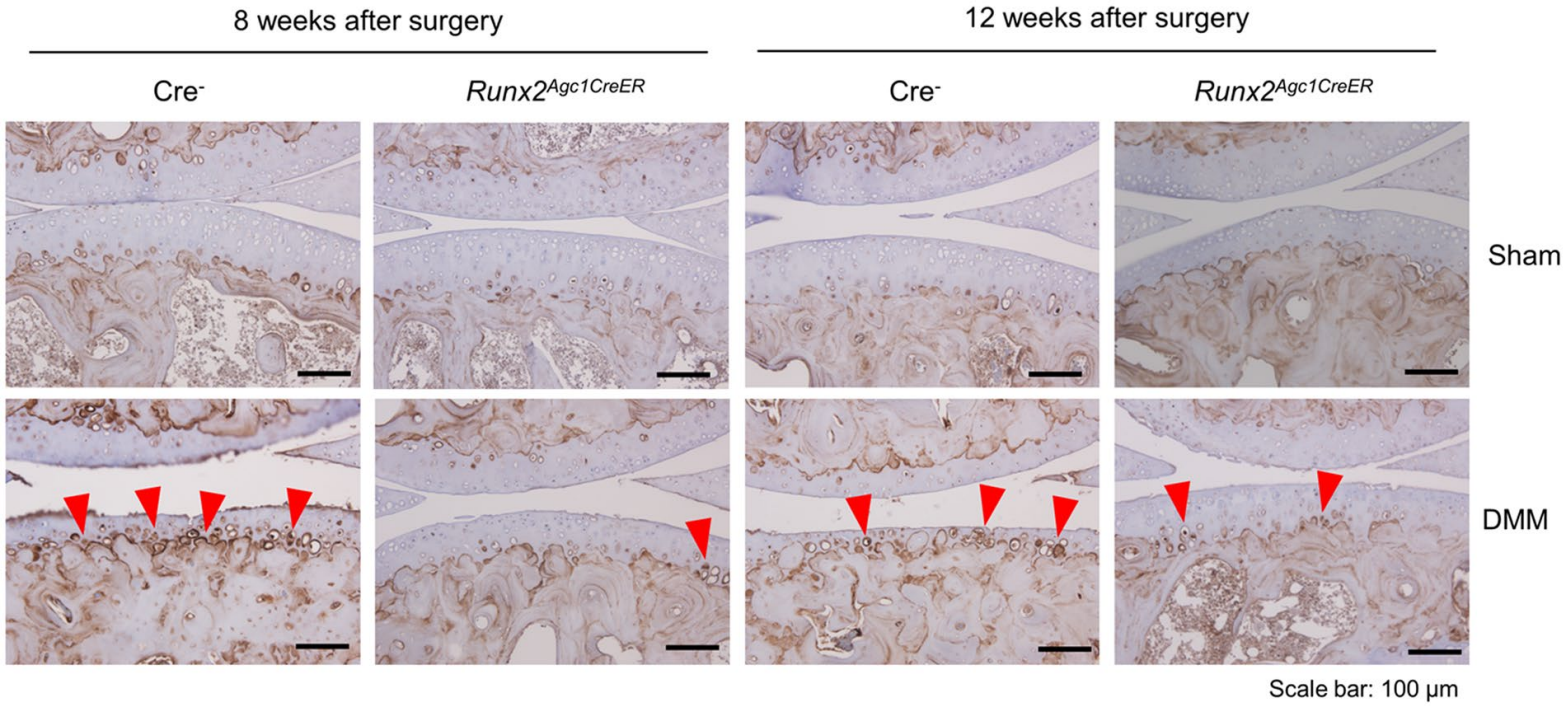
Loss of *Runx2* reversed MMP13 up-regulation induced by DMM surgery. DMM surgery and Sham operation was performed in 12-week-old male Cre-negative and *Runx2* ^*Agc1CreER*^ KO mice. MMP13 expression was analyzed by IHC assay. Increased MMP13 expression was detected and more MMP13-positive cells were located toward articular surface in Cre-negative control mice 8 and 12-weeks after DMM surgery (MMP13-positive cells: red arrowheads). Deletion of *Runx2* significantly down-regulated MMP13 up-regulation induced by DMM (scale bar = 100 μm).

IHC results showed that, MMP13 expression was weak in articular cartilage and was restricted mainly in deep zone (below the tidemark) and adjacent to subchondral bone in Sham operated mice (Fig. 5). However, MMP13 expression was increased in articular cartilage in Cre-negative control mice 8- and 12-weeks after DMM surgery (Fig. 5). MMP13 was expressed not only in deep zone but also in middle and superficial zones of articular cartilage in the mice after DMM surgery (Fig. 5). Since superficial and mid zones of cartilage of Cre-negative mice were no longer present in the mice 12 weeks after DMM surgery, the MMP13 expression at this time point were only observed in deep zone of articular cartilage. The increased MMP13 expression was reduced in both time points 8- and 12-weeks after DMM surgery in *Runx2* ^*Agc1CreER*^ KO mice (Fig. 5).

### The expression of chondrocyte marker genes was reduced in articular chondrocytes derived from *Runx2*^*Agc1CreER*^ KO mice

**Figure 6:**
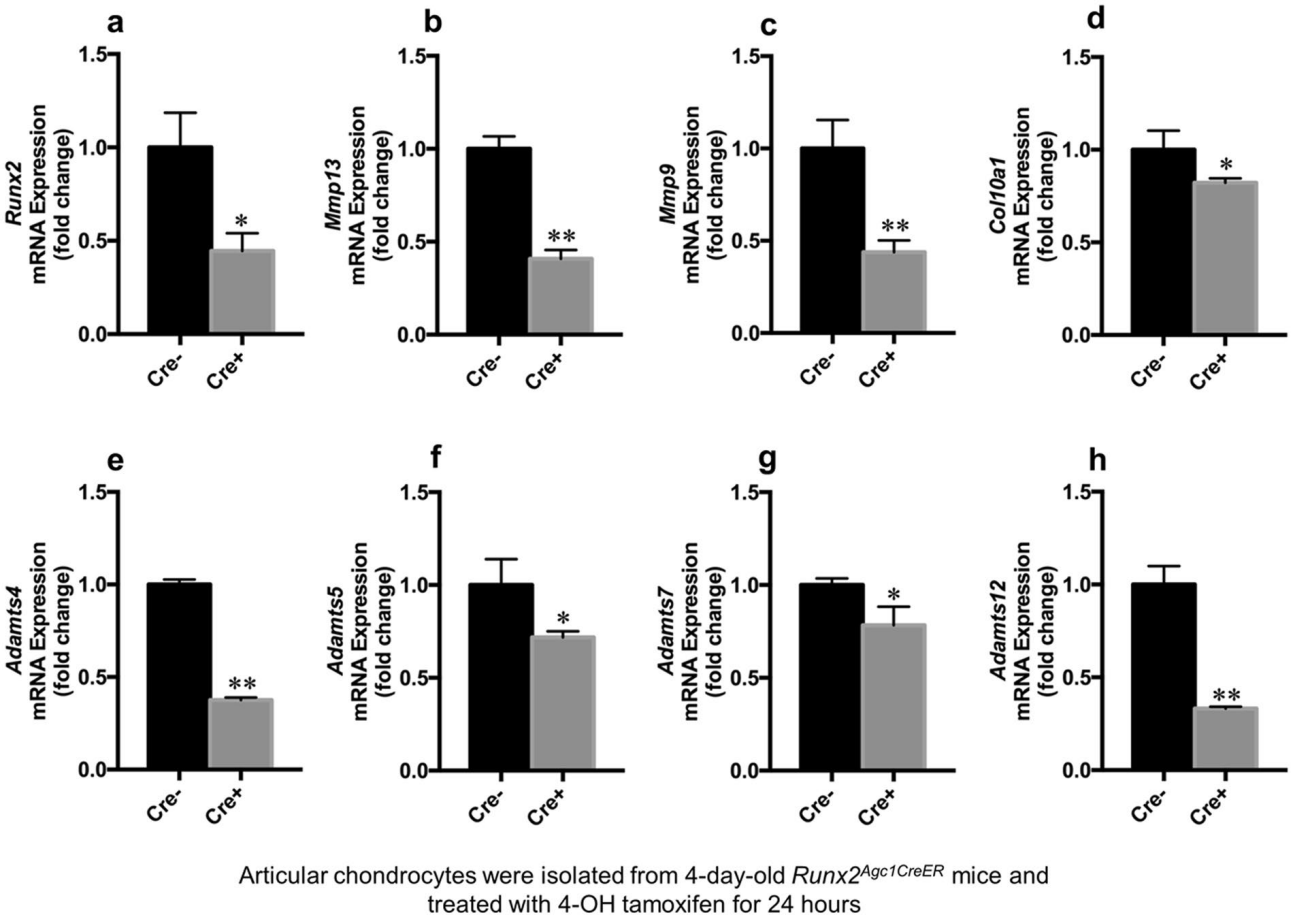
Loss of *Runx2* in chondrocytes isolated from early postnatal mice causes decreased expression of genes encoding to matrix degradation enzymes. (**a**–**h**) Primary articular chondrocytes were isolated from 4-day-old *Runx2* ^*Agc1CreER*^ mice (Cre^+^) and Cre-negative control mice (Cre^−^) and treated with 4-hydroxy tamoxifen (1 μM) for 24 hours, followed by real-time PCR assay. Expression of *Mmp9*, *Mmp13*, *Adamts4*, *Adamts5*, *Adamts7* and *Adamts12* was significantly reduced in *Runx2* deficient chondrocytes (*P < 0.05 and **P < 0.01, Unpaired Student’s *t*-test; n = 3 mice per group).

**Figure 7:**
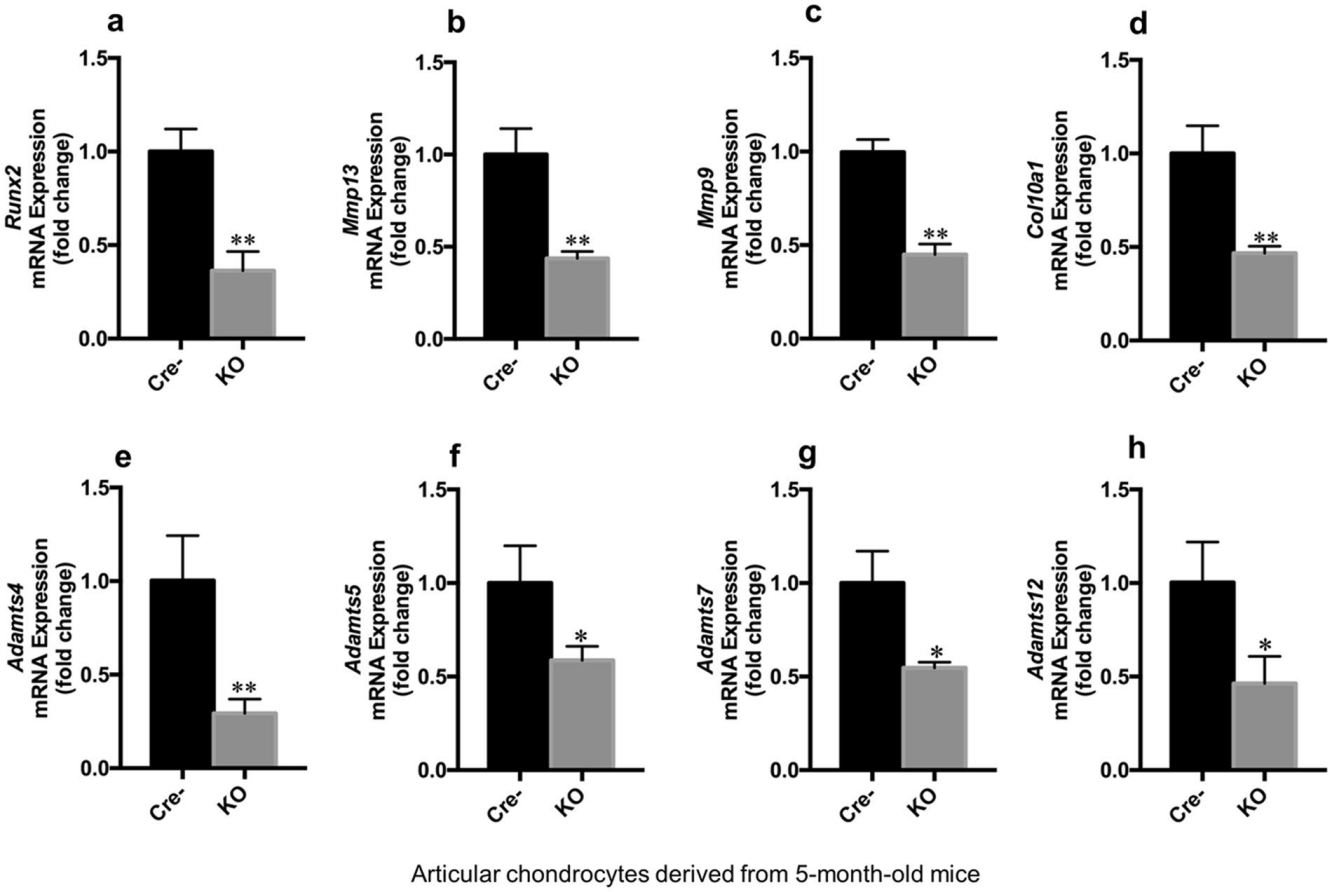
Loss of *Runx2* in adult articular chondrocytes leads to decreased expression of genes encoding to matrix degradation enzymes. (**a**–**h**) Total RNA was extracted from articular chondrocytes isolated from 5-month-old *Runx2* ^*Agc1CreER*^ KO mice (KO) or Cre-negative control mice (Cre^−^) followed by real-time PCR assay. All these mice were administrated with tamoxifen at the two-months of age and received DMM surgery at 12-week-old. Expression of *Mmp9*, *Mmp13*, *Adamts4*, *Adamts5*, *Adamts7* and *Adamts12* was significantly reduced in articular chondrocytes derived from *Runx2* ^*Agc1CreER*^ KO mice (*P < 0.05 and **P < 0.01, unpaired Student’s *t*-test; n = 3 mice per group).

Primary articular chondrocytes were isolated from 4-day-old *Runx2* ^*Agc1CreER*^ mice and control littermates and were treated with 4-hydroxy tamoxifen (1 μM) for 24 hours, followed by real-time PCR assay. The results showed that there was 56% reduction in *Runx2* mRNA expression in articular chondrocytes derived from *Runx2* ^*Agc1CreER*^ mice (Fig. 6a). Expression of *Mmp9* and *Mmp13* was decreased by 56 and 59% in *Runx2* deficient cells (Fig. 6b and c). Similar to *Mmp*, expression of *Adamts* family members was also regulated by Runx2^12, 13^. Expression of *Adamts4* and *Adamts5* was decreased by 62 and 19%, respectively (Fig. 6e and f), and expression of *Adamts7* and *Adamts12* was decreased by 23 and 67%, respectively (Fig. 6g and h) in *Runx2* deficient chondrocytes. To further determine Runx2 regulation of gene expression in articular cartilage *in vivo*, we administrated tamoxifen to 2-month-old *Runx2* ^*Agc1CreER*^ mice and their Cre-negative littermates and isolated mRNA from articular cartilage of Cre-negative and *Runx2* ^*Agc1CreER*^ KO mice. The qRT-PCR data showed the similar gene expression patterns as those observed in the primary articular chondrocytes isolated from 4-day-old *Runx2* ^*Agc1CreER*^ mice treated with 4-OH tamoxifen (Fig. 7a–h).

## Discussion

OA is the most common degenerative joint disorder and a major cause of disability. To investigate the mechanism of the development of OA, several mouse models mimicking human OA were reported in recent years. Among them the well-accepted OA mouse model is DMM-induced OA model. Compared to anterior cruciate ligament transaction (ACLT) model, the OA severity and location of the lesion in DMM model are similar to the lesions observed in aging-related spontaneous mouse model of OA. In addition, DMM model has sufficient sensitivity to show disease modification^3^. The major symptom of OA is the progressive cartilage breakdown and eventually completely loss of articular cartilage^1, 2, 21^. Chondrocytes are the sole cell type in articular cartilage. To better understand the function of specific gene, such as Runx2, in articular cartilage *in vivo*, it is desirable to delete this gene specifically in joint tissues in adult mice^16^.

Aggrecan is a major extracellular matrix (ECM) protein in both growth plate and articular cartilage. A previous study^15^ showed that there was no difference in the localization of *Col2a1* and *Aggrecan* expression in young mice (4–9 weeks old). They express throughout the entire articular cartilage of both medial and lateral tibiae. In contrast to young mice (4–9 weeks old), *Col2a1* expression was not detected in older mice (36–50 weeks old). However, there was strong signal for *Aggrecan* mRNA expression throughout the entire articular cartilage of the medial and lateral tibiae in STR/ort mouse strain. In this study we have used the *Agc1-CreER* mice to delete *Runx2* in articular chondrocytes. The results of frozen sections of *Agc1-CreER; ROSA* ^*mT/mG*^ mice showed that articular chondrocytes can be efficiently targeted by *Agc1-CreER* mice. Furthermore, the results of qRT-PCR and IHC staining showed significantly decreased mRNA and protein levels of Runx2 in *Runx2* ^*Agc1CreER*^ KO mice compared to Cre-negative mice. These findings suggest that Runx2 could be efficiently deleted in articular cartilage in *Runx2* ^*Agc1CreER*^ KO mice.

A recent study showed that *Mmp13* and *Adamts5* up-regulation may be mediated by *Runx2* ^10^. Studies of mutation analysis in the *Mmp13* promoter and the ChIP assays further demonstrated that *Runx2* directly binds to its blinding site at the *Mmp13* promoter in chondrocytes^11^. Recent study also showed that expression of *Adamts7* and *Adamts12* could also be regulated by Runx2 in human chondrocytes^13^.

*Runx2* is a critical transcription factor for chondrocyte maturation^5^ and its role in OA development has not been fully defined. Runx2 expression levels increase in human OA cartilage^6–8^. Additionally, *Runx2* overexpression activated ECM-degrading enzymes (MMP13 and ADAMTS5) through direct interaction with the Runx2 binding sites at the promoters of these genes and through mitogen-activated protein kinase (MAPK) pathway in chondrocytes^10^. A previous study showed that after induction of knee joint instability, *Runx2* global heterozygous KO mice exhibited decreased cartilage destruction and osteophyte formation^14^. The study using *Runx2* heterozygous KO mice did provide important information about the role of Runx2 in OA pathogenesis. However, the *Runx2* deficiency in the heterozygous *Runx2* global KO mice is not chondrocyte-specific and *Runx2* deletion in those mice is not specific at adult stage so the possibilities of embryonic effect (carrying-over to the adult stage) and indirect effect of Runx2 on articular cartilage could not be ruled out in those studies. In the present studies, we have deleted *Runx2* in Aggrecan-expressing chondrocytes and investigated the specific effect of Runx2 in articular chondrocytes at the adult stage. Our studies provided additional and critical information about the role of Runx2 in OA development. Since many genetic OA mouse models showed Runx2 up-regulation, suggesting that Runx2 may be a central molecule mediating downstream target gene expression during OA development, it would be very important to clearly define if Runx2 is indeed required in OA development and progression.

A recent study in our laboratory showed that *Mmp13* and *Adamts5* up-regulation may be mediated by *Runx2* ^11^. Studies of mutation analysis in *Mmp13* promoter and the ChIP assays further demonstrated that *Runx2* directly binds to the OSE2 site at the *Mmp13* promoter^11, 12^. Recent study also showed that expression of *Adamts7* and *Adamts12* could also be regulated by Runx2 in human chondrocytes^13^.

To better understand the role of Runx2 in adult mice during the initiation and progression of OA, in this study we generated *Runx2* ^*Agc1CreER*^ mice by crossing *Runx2* ^*flox/flox*^ mice with *Agc1-CreER* transgenic mice. Tamoxifen was administered to the mice at 8-week-old when growth plate development is basically completed. DMM surgery was performed in both *Runx2* ^*Agc1CreER*^ KO mice and Cre-negative controls at 12-weeks-old. Our results demonstrated that a progressive OA-like phenotype was observed 8- and 12-weeks after DMM surgery in Cre-negative control mice. The evaluation of histology results using the OARSI scoring system^33^ showed that *Runx2* deletion has a protective effect on the DMM-induce OA. Consistently, the histomorphometric analysis of changes in cartilage area at the proximal tibiae showed the similar results. These findings provide evidence showing that *Runx2* deletion has a protective role in the DMM-induced OA.

The critical event in OA development is the progressive loss of articular cartilage. Two major structural components of articular cartilage are collagen and aggrecan. Accordingly, enzymes mainly targeting collagen and aggrecan degradation are MMPs and ADAMTS^2, 34, 35^. A previous study showed that increased expression of *Mmp9* and *Mmp13* was found in the cartilage during OA development^35^. In the present studies, the inhibition of *Mmp9* and *Mmp13* expression was found in *Runx2* deficient chondrocytes, suggesting that *Runx2* is the upstream regulator of *Mmp9* and *Mmp13* expression.

MMP13 is the most potent enzyme among collagenases for degradation of type II collagen^36, 37^. In addition to cleaves type II collagen, it also targets the degradation of aggrecan, collagen types IV and type IX, gelatin, osteonectin, and perlecan in cartilage^38^. The recent studies demonstrated that *Mmp13* may be a downstream target gene of *Runx2* ^11, 12^. In the present studies, we observed the significant reduction of *Mmp13* expression in primary articular chondrocytes of *Runx2* ^*Agc1CreER*^ KO mice. IHC result showed that MMP13 was mainly expressed in the deep zone of articular cartilage, adjacent to subchondral bone and below the tidemark in the Sham operated mice. In mice with DMM surgery, an obvious increase in MMP13 expression was observed in deep zone, as well as in middle and superficial zones of the articular cartilage 8 weeks after DMM surgery. However, superficial and middle zones of the cartilage of Cre-negative mice were no longer present 12 weeks after DMM surgery, so MMP13 expression at this time points were only observed in the deep zone of the articular cartilage. In contrast, the increased MMP13 expression was rescued in *Runx2* ^*Agc1CreER*^ KO mice after DMM surgery.

MMP13 is also a maker of the chondrocyte hypertrophy^39^ and *Runx2* could regulate the *Mmp13* expression in hypertrophic chondrocytes^11, 40^. During OA development, articular chondrocytes undergo hypertrophy leading to extracellular matrix degradation and articular cartilage breakdown^1, 41^. Consistent with these findings, in the present studies, we found that the expanded expression of MMP13 was observed in middle and superficial zones of the cartilage in Cre-negative control mice after DMM surgery, suggesting that chondrocyte hypertrophy is increased in this OA mouse model. Deletion of *Runx2* could significantly inhibit MMP13 expression in *Runx2* ^*Agc1CreER*^ KO mice, even after DMM surgery. The ADADMTS family consists of large members of aggrecanases and they share several distinct modules. Both *Adamts4* and *Adamts5* are responsible for aggrecan degradation in a human model of OA^42^. The previous study showed that *Adamts5* up-regulation may be mediated by *Runx2* in *Tgfbr2* ^*Col2CreER*^ KO mice^12^. The most recent study demonstrated that *Adamts7* and *Adamts12* are also regulated by *Runx2* ^13^. In the present studies, we observed down-regulation of *Adamts 4, 5, 7, 12* mRNA expression in *Runx2* deficient chondrocytes derived from new born *Runx2* ^*Agc1CreER*^ mice treated with 4-OH TM and in articular cartilage of adult *Runx2* ^*Agc1CreER*^ KO mice. *Col10a1* is the most specific hypertrophic chondrocyte maker. The previous studies showed that Runx2 directly targets *Col10a1* transcription through interaction with the cis-enhancing elements^43^. Consistent with this finding, we found a significant down-regulation of *Col10a1* expression in *Runx2* deficient articular chondrocytes.

Runx2 plays a critical role in osteoblast and chondrocyte differentiation in mice and in humans^44, 45^ and is a key transcription factor for chondrocyte hypertrophy and osteoblast differentiation^17^. In chondrocyte-specific *Runx2* KO mice, DMM-induced cartilage degradation was inhibited. A close relationship and a cross-talk may exist between the articular cartilage and subchondral bone. Moreover, cartilage and subchondral bone are in close proximity and soluble proteins produced in the cartilage are likely to be able to move from one compartment to the other. Perturbing cartilage is expected to preferentially affect subchondral bone^46^. This may explain why chondrocyte-specific *Runx2* KO contributes to the inhibition of subchondral bone sclerosis.

In addition to articular cartilage degeneration, subchondral bone sclerosis is also a characteristic of OA^12, 47, 48^. Runx2 plays a critical role in osteoblast and chondrocyte differentiation in mice and in humans^44, 45^ and is a key transcription factor for chondrocyte and osteoblast differentiation^17^. In chondrocyte-specific *Runx2* KO mice, DMM-induced cartilage degradation was inhibited. A close relationship and a cross-talk may exist between the articular cartilage and subchondral bone. Moreover, cartilage and subchondral bone are in close proximity and soluble proteins produced in the cartilage are likely to be able to move from one compartment to the other. Perturbing cartilage is expected to preferentially affect subchondral bone. This may explain why chondrocyte-specific *Runx2* KO contributes to the inhibition of subchondral bone sclerosis.

In summary, we performed chondrocyte-specific *Runx2* deletion in adult *Runx2* KO mice and demonstrated that *Runx2* deletion has chondro-protective effect on DMM-induced OA development and progression. Since we have targeted chondrocytes at adult stage, the potential effects of *Runx2* deletion during embryonic development and indirect effect of Runx2 on growth plate cartilage development have been ruled out in the current studies. Our studies indicate that deletion of *Runx2* in Aggrecan-expressing mature articular chondrocytes prevents DMM-induced OA development. The chondro-protective effect of *Runx2* deletion could be due to the inhibition of genes encoding for multiple matrix degradation enzymes. Our studies suggest that Runx2 could serve as a molecular target for drug development for OA treatment.

## Methods

### DMM-induced OA model

Tamoxifen (Sigma, St. Louis, MO, USA) was administered into 2-month-old *Runx2* ^*Agc1CreER*^ mice and Cre-negative littermates by intraperitoneal (i. p.) injection (1 mg/10 g body weight) for 5 consecutive days^49^. DMM surgery was performed on the right knee of mice to induce knee OA in 12-week-old mice^50^. Sham operation was performed by opening and exposing the structures of the right knee and then closing the skin incision without manipulating the joint tissue on 3-month-old *Runx2* ^*Agc1CreER*^ mice and Cre-negative control mice. Pre- and post-surgery, mice were provided with analgesia (2.5 mg/kg banamine, i. p. injection) every 24 hours for 72 hours and the sutures were removed 10 days after surgery. The right legs were harvested 8 and 12 weeks post-surgery (n = 6–8 in each group), processed, sectioned and stained. The animal protocol of this study has been approved by the IACUC of the Rush University Medical Center and all experimental methods and procedures were carried out in accordance with the approved guidelines.

### The generation of *Runx2*^*Agc1CreER*^ conditional KO mice

*Runx2* ^*flox-neo*^ mice were provided by Dr. Takeshi Takarada^17^ (Okayama University, Japan). Runx2 floxed mice were generated mice carrying a Conditional Runx2 allele with exon 4, which encodes the Runt domain, flanked by loxP sites. *Runx2* ^*flox-neo*^ mice were crossed with FLPe transgenic mice to eliminate the neomycin cassette. To generate *Runx2* ^*Agc1CreER*^ conditional KO mice, *Runx2* ^*flox/flox*^ mice were crossed with *Agc1-CreER* transgenic mice. *Agc1-CreER* transgenic mice were obtained from Jackson laboratories. The resulting *Agc1-CreER; Runx2* ^*flox/flox*^ (*Runx2* ^*Agc1CreER*^) mice were administered with tamoxifen (1 mg/10 g body weight/day, i. p. injection, for 5 days) at age two-month-old and were sacrificed at ages 5 or 6 months (8 and 12 weeks post DMM surgery) for histologic analysis. Cre-negative littermates were used as controls.

### Cre recombination efficiency

To determine whether *Agc1-CreER* mice could target articular chondrocytes efficiently in adult mice, *Agc1-CreER* transgenic mice were bred with *ROSA* ^*mT/mG*^ reporter mice^32^ (obtained from Jackson Laboratories). Tamoxifen was administered into 2-month-old mice. Mice were sacrificed at age of 3 months. Histologic sections were analyzed using a fluorescence microscope.

### Micro-computed tomography (μCT)

Prior to histologic processing, we evaluated formalin-fixed mouse legs by μCT using a μCT-35 cone-beam scanner (Scanco Medical) with a 55 kVp source and a 145 μAmp current. We scanned the mouse legs at a resolution of 12 μm. Morphometric analysis was performed on 50 slices extending proximally, beginning with the first slice in which the tibia condyles had fully merged. The subchondral bone was segmented from the cortical shell manually on key slices using a contouring tool, and the contours were morphed automatically to segment the trabecular bone on all slices. The morphometry was reconstructed and analyzed.

### Histology and immunohistochemistry

Knee joint tissues were fixed in 4% paraformaldehyde for 48 hours, decalcified with 10% formic acid (commercially-available decalcification solution) for ten days, dehydrated with graded ethanol, and embedded in paraffin. Serial mid-sagittal sections (3-μm thick) were cut and stained with Alcian blue/hematoxylin and eosin (AB/H&E) for morphologic analysis^51^. Histomorphometric measurements were performed with OsteoMeasure software (OsteoMetrics, Inc., Atlanta, GA, USA). AB/H&E-stained areas were outlined on projected images of each histologic section to determine articular cartilage area^12^. IHC was performed on the 3-μm thick tissue sections and sections were baked at 60 °C overnight. Slides were then deparaffinized, rehydrated, and washed twice in dH_2_O for 5 minutes each. The antigen retrieval was performed with Antigen Unmasking solution (Vector Laboratories, H-3300) in 95 °C for 10 minutes. Slides were then quenched in 3% hydrogen peroxide for 10 minutes at room temperature. Slides were incubated with 0.5% Triton X-100 (Sigma-Aldrich, 9002-93-1) for 1 hour, and washed with PBS for 3 times and then blocked with Avidin/Biotin Blocking Kit (Invitrogen, 004303). Slides were then washed again with PBS for 3 times and then blocked with the blocking serum at 10% normal goat serum (Vector Laboratories, S-1000) in 1% BSA for 30 minutes at room temperature. Slides were then incubated with primary antibodies against MMP13 (Mouse anti-Human, MAB 13424, 1:100 dilution) or Runx2 (Mouse IgG, MBL, D130-3, 1:200 dilution) at 4 °C overnight. On the second day, secondary biotinylated goat anti-mouse antibody (Vector Laboratories, BA-9200) was added for 30 minutes, followed by incubation with VECTASTAIN Elite ABC HRP Kit (Vector Laboratories, PK-6100) for 30 minutes. Positive staining was detected by ImmPACT DAB Peroxidase (HRP) Substrate (Vector Laboratories, PK-6100). Slides were then counterstained with CAT Hematoxylin (Biocare Medical, CATHE-GL), dehydrated with graded ethanol and cleared with 3 changes of Xylene and then coversliped.

### Grading of cartilage structure

Histology sections of knee joint (tibia, sagittal view) were stained with Alcian blue/Orange G and graded by two blinded observers based on the scoring system developed by Glasson *et al*.^33^. In brief, each section was assigned a grade 0–6: 0, normal cartilage; 0.5, loss of Safranin O staining without structural changes; 1, small fibrillations without loss of cartilage; 2, vertical clefts down to the layer below the superficial layer; 3–6, vertical clefts or erosion to the calcified cartilage (<25% (grade 3), 25–50% (grade 4), 50–75% (grade 5) and >75% (grade 6) of the articular surface is affected)^33^. The maximal score was used to represent severity of the OA progression of each mouse.

### Cell culture and real-time polymerase chain reaction (PCR) analysis

Primary articular chondrocytes were isolated from articular cartilage of 4-day-old neonatal mice, as described previously^52^. The isolated cells were treated with 4-OH tamoxifen (1 μM) for 24 hours. Total mRNA was extract with Trizol (Invitrogen Life Technologies, CA, USA). 1 μg total RNA was used to synthesize complementary DNA (cDNA) using an iScripts cDNA Synthesis kit (Quanta Biosciences, MD, USA). Real-time PCR amplification was performed using specific primers of genes encoding for matrix degradation enzymes and a SYBR Green real-time PCR kit (Quanta Biosciences, MD, USA). The primer names and sequences were listed in Table 1. Data were collected from cells of 3 independent mice (n = 3).

**Table 1:** The names of sequences of primers used in this project.

Genes	Primer sequence (forward primers)	Primer sequence (reverse primers)
*Runx2*	GACTGTGGTTACCGTCATGGC	ACTTGGTTTTTCATAACAGCGGA
*Mmp9*	GCAGAGGCATACTTGTACCG	TGATGTTATGATGGTCCCACTTG
*Mmp13*	CTTCTTCTTGTTGAGCTGGACTC	CTGTGGAGGTCACTGTAGACT
*Adamts4*	ATGGCCTCAATCCATCCCAG	GCAAGCAGGGTTGGAATCTTTG
*Adamts5*	GGAGCGAGGCCATTTACAAC	CGTAGACAAGGTAGCCCACTTT
*Adamts7*	GCAGGCTTCGTCTGCTTTCTA	GCCATCAGATAAGGGTTGGTGG
*Adamts12*	GACCCGAGGCAAGAACATTTT	CCCAGTTGACCGTCAGATTGA
*Col10a1*	TTCTGCTGCTAATGTTCTTGACC	GGGATGAAGTATTGTGTCTTGGG
*Actin*	GGCTGTATTCCCCTCCATCG	CCAGTTGGTAACAATGCCATGT

### Statistical analysis

Data are presented as the mean ± SD. For experiments comparing two groups of data, unpaired Student *t*-test was performed. For data that multiple groups are involved, one-way analysis of variance (ANOVA) was performed followed by Turkey’s post-hoc test. P values less than 0.05 were considered significant.

## References

[1] Goldring MB, Goldring SR (2007). Osteoarthritis. J Cell Physiol.

[2] Wang M (2011). Recent progress in understanding molecular mechanisms of cartilage degeneration during osteoarthritis. Ann N Y Acad Sci.

[3] Glasson SS, Blanchet TJ, Morris EA (2007). The surgical destabilization of the medial meniscus (DMM) model of osteoarthritis in the 129/SvEv mouse. Osteoarthritis Cartilage.

[4] Chen CG, Thuillier D, Chin EN, Alliston T (2012). Chondrocyte-intrinsic Smad3 represses Runx2-inducible matrix metalloproteinase 13 expression to maintain articular cartilage and prevent osteoarthritis. Arthritis Rheum.

[5] Yoshida CA (2004). Runx2 and Runx3 are essential for chondrocyte maturation, and Runx2 regulates limb growth through induction of Indian hedgehog. Genes Dev.

[6] Wang X (2004). Regulation of MMP-13 expression by RUNX2 and FGF2 in osteoarthritic cartilage. Osteoarthritis Cartilage.

[7] Zhong, L., Huang, X., Karperien, M. & Post, J.N. Correlation between Gene Expression and Osteoarthritis Progression in Human. *Int J Mol Sci* **17** (2016). 10.3390/ijms17071126 PMC4964500 27428952

[8] Hasegawa A (2013). Cellular and extracellular matrix changes in anterior cruciate ligaments during human knee aging and osteoarthritis. Arthritis Res Ther.

[9] Kadri A (2010). Inhibition of bone resorption blunts osteoarthritis in mice with high bone remodelling. Ann Rheum Dis.

[10] Tetsunaga T (2011). Regulation of mechanical stress-induced MMP-13 and ADAMTS-5 expression by RUNX-2 transcriptional factor in SW1353 chondrocyte-like cells. Osteoarthritis Cartilage.

[11] Wang M (2012). Conditional activation of β-catenin signaling in mice leads to severe defects in intervertebral disc tissue. Arthritis Rheum.

[12] Shen J (2013). Deletion of the transforming growth factor beta receptor type II gene in articular chondrocytes leads to a progressive osteoarthritis-like phenotype in mice. Arthritis Rheum.

[13] Ji Q (2016). miR-105/Runx2 axis mediates FGF2-induced ADAMTS expression in osteoarthritis cartilage. J Mol Med (Berl).

[14] Kamekura S (2006). Contribution of runt-related transcription factor 2 to the pathogenesis of osteoarthritis in mice after induction of knee joint instability. Arthritis Rheum.

[15] Chambers MG, Kuffner T, Cowan SK, Cheah KS, Mason RM (2002). Expression of collagen and aggrecan genes in normal and osteoarthritic murine knee joints. Osteoarthritis Cartilage.

[16] Henry SP (2009). Generation of aggrecan-CreERT2 knockin mice for inducible Cre activity in adult cartilage. Genesis.

[17] Takarada T (2013). An analysis of skeletal development in osteoblast-specific and chondrocyte-specific runt-related transcription factor-2 (Runx2) knockout mice. J Bone Miner Res.

[18] Bau B (2002). Relative messenger RNA expression profiling of collagenases and aggrecanases in human articular chondrocytes *in vivo* and *in vitro*. Arthritis Rheum.

[19] Neuhold LA (2001). Postnatal expression in hyaline cartilage of constitutively active human collagenase-3 (MMP-13) induces osteoarthritis in mice. J Clin Invest.

[20] Roach HI (2005). Association between the abnormal expression of matrix-degrading enzymes by human osteoarthritic chondrocytes and demethylation of specific CpG sites in the promoter regions. Arthritis Rheum.

[21] Wang M (2013). MMP13 is a critical target gene during the progression of osteoarthritis. Arthritis Res Ther.

[22] Ducy P, Karsenty G (1995). Two distinct osteoblast-specific cis-acting elements control expression of a mouse osteocalcin gene. Mol Cell Biol.

[23] Ducy P, Zhang R, Geoffroy V, Ridall AL, Karsenty G (1997). Osf2/Cbfa1: a transcriptional activator of osteoblast differentiation. Cell.

[24] Jimenez MJ (1999). Collagenase 3 is a target of Cbfa1, a transcription factor of the runt gene family involved in bone formation. Mol Cell Biol.

[25] Winchester SK, Selvamurugan N, D’Alonzo RC, Partridge NC (2000). Developmental regulation of collagenase-3 mRNA in normal, differentiating osteoblasts through the activator protein-1 and the runt domain binding sites. J Biol Chem.

[26] Hess J, Porte D, Munz C, Angel P (2001). AP-1 and Cbfa/runt physically interact and regulate parathyroid hormone-dependent MMP13 expression in osteoblasts through a new osteoblast-specific element 2/AP-1 composite element. J Biol Chem.

[27] Mengshol JA, Vincenti MP, Brinckerhoff CE (2001). IL-1 induces collagenase-3 (MMP-13) promoter activity in stably transfected chondrocytic cells: requirement for Runx-2 and activation by p38 MAPK and JNK pathways. Nucleic Acids Res.

[28] Porte D (1999). Both AP-1 and Cbfa1-like factors are required for the induction of interstitial collagenase by parathyroid hormone. Oncogene.

[29] D’Alonzo RC, Selvamurugan N, Karsenty G, Partridge NC (2002). Physical interaction of the activator protein-1 factors c-Fos and c-Jun with Cbfa1 for collagenase-3 promoter activation. J Biol Chem.

[30] Jimenez MJ (2001). A regulatory cascade involving retinoic acid, Cbfa1, and matrix metalloproteinases is coupled to the development of a process of perichondrial invasion and osteogenic differentiation during bone formation. J Cell Biol.

[31] Nishimura R (2012). Osterix regulates calcification and degradation of chondrogenic matrices through matrix metalloproteinase 13 (MMP13) expression in association with transcription factor Runx2 during endochondral ossification. J Biol Chem.

[32] Muzumdar MD, Tasic B, Miyamichi K, Li L, Luo L (2007). A global double-fluorescent Cre reporter mouse. Genesis.

[33] Glasson SS, Chambers MG, Van Den Berg WB, Little CB (2010). The OARSI histopathology initiative - recommendations for histological assessments of osteoarthritis in the mouse. Osteoarthritis Cartilage.

[34] Davidson RK (2006). Expression profiling of metalloproteinases and their inhibitors in synovium and cartilage. Arthritis Res Ther.

[35] Kevorkian L (2004). Expression profiling of metalloproteinases and their inhibitors in cartilage. Arthritis Rheum.

[36] Knauper V, Lopez-Otin C, Smith B, Knight G, Murphy G (1996). Biochemical characterization of human collagenase-3. J Biol Chem.

[37] Reboul P, Pelletier JP, Tardif G, Cloutier JM, Martel-Pelletier J (1996). The new collagenase, collagenase-3, is expressed and synthesized by human chondrocytes but not by synoviocytes. A role in osteoarthritis. J Clin Invest.

[38] Shiomi T, Lemaitre V, D’Armiento J, Okada Y (2010). Matrix metalloproteinases, a disintegrin and metalloproteinases, and a disintegrin and metalloproteinases with thrombospondin motifs in non-neoplastic diseases. Pathol Int.

[39] Yang L, Tsang KY, Tang HC, Chan D, Cheah KS (2014). Hypertrophic chondrocytes can become osteoblasts and osteocytes in endochondral bone formation. Proc Natl Acad Sci USA.

[40] Hirata M (2012). C/EBPbeta and RUNX2 cooperate to degrade cartilage with MMP-13 as the target and HIF-2alpha as the inducer in chondrocytes. Hum Mol Genet.

[41] Felson DT (2006). Clinical practice. Osteoarthritis of the knee. N Engl J Med.

[42] Verma P, Dalal K (2011). ADAMTS-4 and ADAMTS-5: key enzymes in osteoarthritis. J Cell Biochem.

[43] Li F (2011). Runx2 contributes to murine Col10a1 gene regulation through direct interaction with its cis-enhancer. J Bone Miner Res.

[44] Lee B (1997). Missense mutations abolishing DNA binding of the osteoblast-specific transcription factor OSF2/CBFA1 in cleidocranial dysplasia. Nat Genet.

[45] Mundlos S (1997). Mutations involving the transcription factor CBFA1 cause cleidocranial dysplasia. Cell.

[46] Findlay DM, Kuliwaba JS (2016). Bone-cartilage crosstalk: a conversation for understanding osteoarthritis. Bone Res.

[47] Suri S, Walsh DA (2012). Osteochondral alterations in osteoarthritis. Bone.

[48] Zhen G (2013). Inhibition of TGF-beta signaling in mesenchymal stem cells of subchondral bone attenuates osteoarthritis. Nat Med.

[49] Chen M, Li S, Xie W, Wang B, Chen D (2014). Col2CreER(T2), a mouse model for a chondrocyte-specific and inducible gene deletion. Eur Cell Mater.

[50] Sampson ER (2011). Establishment of an index with increased sensitivity for assessing murine arthritis. J Orthop Res.

[51] Shu B (2011). BMP2, but not BMP4, is crucial for chondrocyte proliferation and maturation during endochondral bone development. J Cell Sci.

[52] Gosset M, Berenbaum F, Thirion S, Jacques C (2008). Primary culture and phenotyping of murine chondrocytes. Nat Protoc.

